# Association Between Alkaline Phosphatase and Clinical Outcomes in Patients With Spontaneous Intracerebral Hemorrhage

**DOI:** 10.3389/fneur.2021.677696

**Published:** 2021-08-30

**Authors:** Sijia Li, Wenjuan Wang, Qian Zhang, Yu Wang, Anxin Wang, Xingquan Zhao

**Affiliations:** ^1^Department of Neurology, Beijing Tiantan Hospital, Capital Medical University, Beijing, China; ^2^China National Clinical Research Center for Neurological Diseases, Beijing, China; ^3^Research Unit of Artificial Intelligence in Cerebrovascular Disease, Chinese Academy of Medical Sciences, Beijing, China

**Keywords:** alkaline phosphatase, spontaneous intracerebral hemorrhage, clinical outcomes, hemorrhagic, stroke

## Abstract

**Background:** Spontaneous intracerebral hemorrhage (ICH) is associated with high rates of mortality and morbidity. Alkaline phosphatase (ALP) is related to increased risk of cardiovascular events and is also closely associated with adverse outcomes after ischemic or hemorrhagic stroke. However, there are limited data about the effect of ALP on clinical outcomes after ICH. Therefore, we aimed to investigate the relationship between serum ALP level and prognosis in ICH patients.

**Methods:** From January 2014 to September 2016, 939 patients with spontaneous ICH were enrolled in our study from 13 hospitals in Beijing. Patients were categorized into four groups based on the ALP quartiles (Q1, Q2, Q3, Q4). The main outcomes were 30-day, 90-day, and 1-year poor functional outcomes (modified Rankin Scale score of 3–6). Multivariable logistic regression and interaction analyses were performed to evaluate the relationships between ALP and clinical outcomes after ICH.

**Results:** In the logistic regression analysis, compared with the third quartile of ALP, the adjusted odds ratios of the Q1, Q2, and Q4 for 30-day poor functional outcome were 1.31 (0.80–2.15), 1.16 (0.71–1.89), and 2.16 (1.32–3.55). In terms of 90-day and 1-year poor functional outcomes, the risks were significantly higher in the highest quartile of ALP compared with the third quartile after adjusting the confounding factors [90-day: highest quartile OR = 1.86 (1.12–3.10); 1-year: highest quartile OR = 2.26 (1.34–3.80)]. Moreover, there was no significant interaction between ALP and variables like age or sex.

**Conclusions:** High ALP level (>94.8 U/L) was independently associated with 30-day, 90-day, and 1-year poor functional outcomes in ICH patients. Serum ALP might serve as a predictor for poor functional outcomes after ICH onset.

## Introduction

Spontaneous intracerebral hemorrhage (ICH) is one of the most common stoke subtypes (1, 2), resulting in striking morbidity and mortality (1, 2). The age-adjusted incidence of ICH for individuals ≥55 years of age in China was generally higher than that in Western countries (3, 4). China Stroke Statistics 2019 reported that for ICH patients, in-hospital mortality was 19.5% (5). Given that there are limited therapeutic strategies in ICH patients (6), early identification and management of the risk factors for the poor prognosis are urgently needed.

Alkaline phosphatase (ALP), first discovered in 1923 (7), is an enzyme that catalyzes the hydrolysis of pyrophosphate from nucleotides and proteins (8, 9). Serum ALP has been implicated to regulate the balance between promoters and inhibitors of mineralization and enhanced vascular calcification (10, 11). Previous studies demonstrated that elevated ALP was associated with all-cause mortality and subsequent cardiovascular disease in myocardial infarction survivors, clinic populations, and general populations (9). Moreover, it has been reported that ALP could predict mortality, functional outcome, and stroke recurrence, especially in those with ischemic stroke (11–17). However, the role of ALP on clinical outcomes of ICH patients has not been fully interpreted in previous studies conducted in a single center with relatively small cohort, and the underlying mechanism remains unclear. Some suggested that vascular dysfunction and atherosclerotic process may play a crucial role in ICH clinical outcomes (16, 18).

Therefore, in this study, we aimed to investigate the association between serum ALP level and clinical outcomes in patients with spontaneous ICH.

## Materials and Methods

### Study Design and Population

The study was a multicenter, prospective, observational cohort study, conducted in 13 hospitals in Beijing from January 2014 to September 2016. The study was carried out in compliance with the guidelines from the World Medical Association Declaration of Helsinki and was approved by the Institutional Review Board (IRB) of Beijing Tiantan Hospital. The ethics committee(s) approved consent by proxy in the ethics statement. Written informed consent was obtained from patients or their legally authorized representatives. Participating centers collected data and submitted it online to the coordinating center of Beijing Tiantan Hospital.

The inclusion criteria were as follows: (1) ICH patients diagnosed by the WHO standard and confirmed by CT scan, (2) first-ever acute-onset ICH, (3) age ≥ 18 years old, and (4) arriving at the hospital within 72 h after symptom onset.

**Figure 1:**
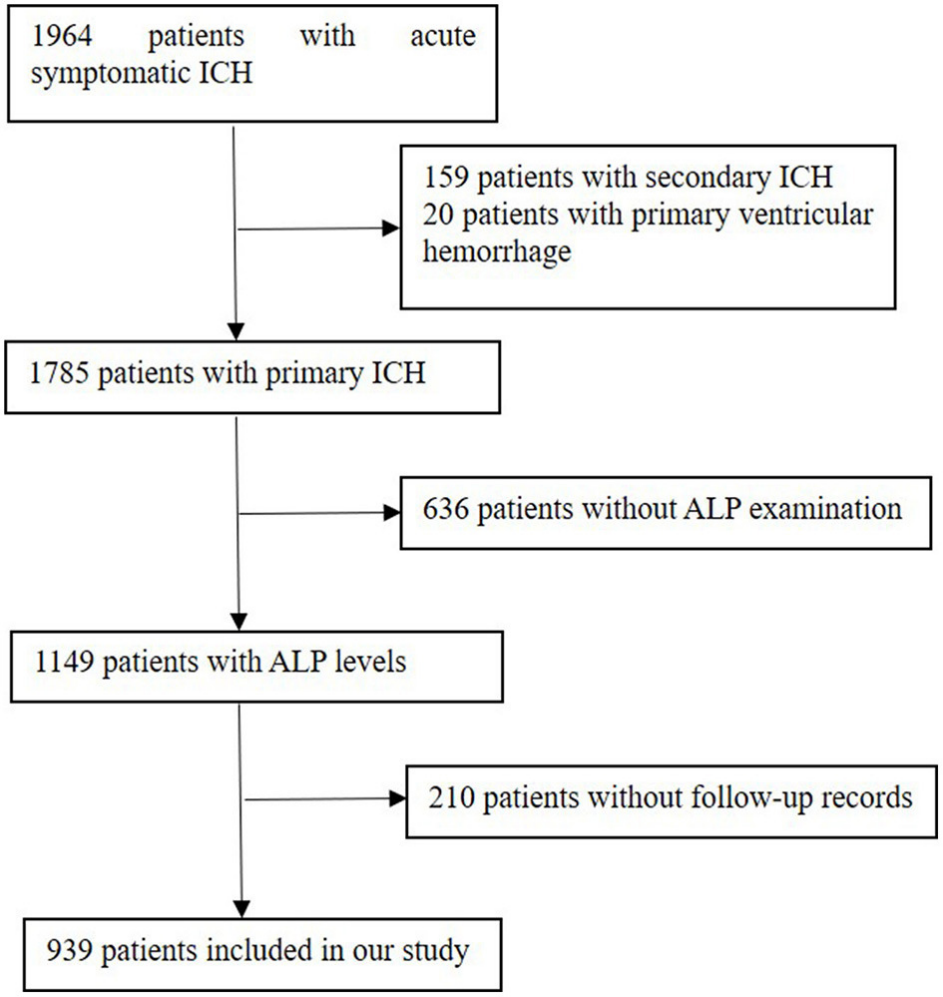
Flow diagram of study patients. ICH, intracerebral hemorrhage; ALP, alkaline phosphatase.

The exclusion criteria were patients complicated with major comorbidities or late-stage diseases, which referred to liver failure (Child–Pugh score C), end-stage kidney disease [estimated glomerular filtration rate (eGFR) is <15 ml/min per 1.73 m^2^], heart failure with reduced ( ≤ 40%) left ventricular ejection fraction, and malignant tumor with a life expectancy of <3 months. There were 1,964 ICH patients enrolled in our database. The additional exclusion criteria of this analysis were as follows: (1) patients with secondary ICH, which attribute to aneurysms, cerebrovascular malformations, cerebral venous thrombosis, trauma, tumor, or hemorrhagic transformation of ischemic stroke; (2) patients with primary ventricular hemorrhage; and (3) lack of serum ALP concentration and patients without follow-up records. As a result, 939 patients were finally enrolled in this study (Figure 1).

### Baseline Information

Baseline information including age, sex, medical history (hypertension, diabetes mellitus, dyslipidemia, and cerebral infarction), health habits (smoking and alcohol consumption), and concomitant medications were collected using standard questionnaires. Hypertension was defined as a self-reported history, a systolic blood pressure ≥ 140 mmHg, or diastolic blood pressure ≥ 90 mmHg at baseline or taking any antihypertensive medicine. Diabetes mellitus was noted as a self-reported history, fasting blood glucose level ≥ 7.0 mmol/L at baseline, or treated with hypoglycemic drug or insulin. Dyslipidemia was defined as a self-reported history, current use of lipid-lowering agents, or a total cholesterol level ≥ 6.22 mmol/L or triglyceride ≥ 2.26 mmol/L or low-density lipoprotein ≥ 4.14 mmol/L at baseline. Smoking was documented when a patient smoked at least one cigarette per day for over a year. Alcohol consumption was defined as an intake of at least 80 g of liquor a day for more than a year.

Neurological deficit was assessed using the Glasgow Coma Scale (GCS) and the National Institutes of Health Stroke Scale (NIHSS) at admission. We also recorded the hematoma location (lobar, basal ganglia, thalamus, brainstem, cerebellum) and volume (ABC/2 method) (19) based on the initial CT scan, which was completed within 24 h after admission.

### ALP Testing and Other Laboratory Examinations

Blood samples were collected from an antecubital vein the next morning after overnight fasting for at least 8 h, and serum ALP levels were measured using unfrozen samples by an automated enzymatic method at each qualified study center.

Other laboratory examinations, including alanine aminotransferase (ALT), aspartate aminotransferase (AST), fasting blood glucose (FBG), total cholesterol (TC), triglyceride (TG), low-density lipoprotein cholesterol (LDL-C), and high-density lipoprotein cholesterol (HDL-C), were also measured during admission. eGFR was calculated using the Chronic Kidney Disease Epidemiology Collaboration creatinine equation with an adjusted coefficient of 1.1 for the Asian population (20).

### Follow-Up Information and Clinical Outcome

Telephone interviews were carried out for all the patients at 30 days, 90 days, and 1 year separately after ICH onset. Functional outcomes of the included patients were assessed using the modified Rankin Scale score (mRS) by trained research interviewers. A structured interview protocol was used in all telephone follow-ups, and the interviewers were blinded to the baseline characteristics and prognostic factors at each follow-up. For patients who were not reached at the first telephone interview, we made telephone follow-up interviews weekly until three missed calls were recorded; at this point, the follow-up was considered as lost. The clinical outcome was defined as 30-day, 90-day, and 1-year poor functional outcomes. Poor functional outcome, namely, death or disability, was defined as mRS of 3–6.

### Statistics Analysis

The statistical analysis was conducted using the SAS software (version 9.4; SAS Institute, Cary, NC, USA). All the participants were categorized into four groups according to quartiles of serum ALP levels (Q1, Q2, Q3, Q4). Continuous variables were expressed as means ± standard deviation (SD) or medians (interquartile range, IQR) and were compared by variance analysis. Categorical variables were presented as numbers (proportions) and were compared using chi-squared tests. A multivariate logistic regression model analysis was performed to estimate the association between ALP levels and clinical outcomes. Variables associated with adverse clinical outcomes of ICH for theoretical considerations or variables based on differences in baseline characteristics between different ALP levels were finally entered in the multivariable models. These included age, sex, alcohol, hypertension, diabetes mellitus, dyslipidemia, history of cerebral infarction, prior antiplatelet use, prior anticoagulant use, body mass index (BMI), systolic blood pressure, diastolic blood pressure, GCS score, NIHSS score, location of hematoma, hematoma volume, ALT, AST, eGFR, fasting blood glucose, surgical treatment, and whether breaking into ventricle or subarachnoid. Odd ratios (ORs) and 95% confidence intervals (CIs) were calculated for each group with the third quartile as reference for ALP. All tests of significance were two-tailed, and a *p*-value <0.05 was considered to be statistically significant.

## Results

### Baseline Characteristics

Baseline characteristics by quartiles of ALP are provided in Table 1. The study included 939 patients, the mean age was 58.7 years old, 69.8% (655/939) were men, and 96.4% of patients were Han. The patients from the higher quartiles of ALP were more likely to have higher ALT and AST. However, no significant differences were observed in age, sex, BMI, smoking and drinking status, hypertension, diabetes mellitus, dyslipidemia, and history of cerebral infarction between different levels of ALP. In addition, there was no difference in blood pressure, GCS score, NIHSS score, location and volume of hematoma, other laboratory results, and whether underwent surgical treatment between groups. The basic characteristics between included and excluded patients in our study are shown in Supplementary Table 1. Patients excluded in our study tend to be younger and have lower proportions of hypertension, diabetes mellitus, dyslipidemia, and surgical treatment, as well as have relatively higher ALT, AST, and eGFR. We also discovered that in the excluded group, the GCS score was significantly lower, while the NIHSS score and hematoma volume were significantly higher.

**Table 1:** Baseline characteristics of the participants according to the quartiles of ALP levels.

	**Overall (*n* = 939)**	**Q1 (≤58.0)**	**Q2 (58.0–75.0)**	**Q3 (75.0–94.8)**	**Q4 (>94.8)**	***p*-value**
Male, *n* (%)	655 (69.8)	170 (72.3)	159 (68.0)	169 (72.2)	157 (66.5)	0.40
Age (years)	58.7 ± 13.2	60.5 ± 13.6	58.4 ± 13.5	58.0 ± 13.0	57.9 ± 12.8	0.18
Ethnic Han, *n* (%)	905 (96.4)	215 (94.7)	227 (97.0)	220 (97.4)	243 (96.4)	0.44
Current smoking, *n* (%)	307 (32.7)	84 (35.7)	66 (28.2)	76 (32.5)	81 (34.3)	0.33
Alcohol, *n* (%)	352 (37.5)	82 (34.90)	81 (34.6)	96 (41.0)	93 (39.4)	0.37
Hypertension, *n* (%)	896 (95.4)	221 (94.0)	223 (95.3)	227 (97.0)	225 (95.3)	0.50
Diabetes mellitus, *n* (%)	323 (34.4)	84 (35.7)	78 (33.3)	84 (35.9)	77 (32.6)	0.83
Dyslipidemia, *n* (%)	308 (32.8)	73 (31.1)	72 (30.8)	82 (35.0)	81 (34.3)	0.67
History of cerebral infarction, *n* (%)	135 (14.4)	32 (13.6)	39 (16.7)	34 (14.5)	30 (12.7)	0.65
Prior antiplatelet use, *n* (%)	152 (16.2)	34 (14.5)	37 (15.8)	44 (18.8)	37 (15.7)	0.62
Prior anticoagulant use, *n* (%)	11 (1.2)	3 (1.3)	4 (1.7)	1 (0.4)	3 (1.3)	0.63
BMI	25.6 ± 3.6	25.5 ± 3.8	25.6 ± 3.8	26.0 ± 3.4	25.3 ± 3.3	0.16
SBP (mmHg)	163.5 (149.0–185.0)	163.5 (147.0–188.0)	163.5 (148.0–180.0)	163.3 (150.0–185.0)	163.5 (150.0–185.0)	0.80
DBP (mmHg)	97.0 (84.0–109.0)	98.0 (86.0–109.0)	96.0 (80.0–106.0)	96.0 (83.0–108.0)	97.0 (84.5–110.0)	0.33
GCS score	14 (11–15)	14 (9–15)	14 (10–15)	14 (11–15)	14 (10–15)	0.36
NIHSS score	10 (3–16)	10 (3–19)	10 (3–16)	10 (3–15)	9 (3–16)	0.55
Location of hematoma, *n* (%)						0.30
Lobar	161 (17.2)	40 (17.0)	39 (16.7)	41 (17.5)	41 (17.4)	
Deep	585 (62.3)	154 (65.5)	143 (61.1)	152 (65.0)	136 (57.6)	
Infratentorial	93 (9.9)	26 (11.1)	23 (9.8)	18 (7.7)	26 (11.0)	
Hematoma volume (ml)	14.6 (6.0–30.0)	14.6 (5.5–27.0)	15.1 (8.0–33.3)	14.6 (6.0–31.4)	13.5 (6.0–28.8)	0.31
Break into ventricle, *n* (%)	311 (33.1)	77 (32.8)	68 (29.1)	82 (35.0)	84 (35.6)	0.42
Break into subarachnoid, *n* (%)	96 (10.2)	27 (11.5)	24 (10.3)	20 (8.6)	25 (10.6)	0.76
ALT (U/L)	21.3 (14.0–31.0)	18.0 (11.0–26.0)	21.0 (14.0–29.2)	23.0 (15.0–33.0)	24.5 (17.0–35.6)	<0.0001
AST (U/L)	21.0 (17.0–27.4)	20.0 (16.0–25.0)	20.7 (16.4–26.9)	21.0 (17.0–26.0)	24.0 (19.0–31.0)	<0.0001
eGFR (ml/min)	54.4 (50.7–58.2)	53.7 (49.8–58.0)	54.6 (51.0–58.0)	54.7 (51.1–58.5)	54.7 (51.1–58.4)	0.18
FBG (mmol/L)	5.9 (5.0–7.1)	5.9 (5.0–7.2)	5.9 (5.1–7.0)	5.9 (5.1–7.2)	5.9 (4.8–6.8)	0.54
Surgical treatment, *n* (%)	200 (21.3)	55 (23.4)	45 (19.2)	55 (23.5)	45 (19.1)	0.46

*Continuous variables are expressed as means ± (SD) or medians (IQR) as appropriate.*

*ALP, alkaline phosphatase; BMI, body mass index; SBP, systolic blood pressure; DBP, diastolic blood pressure; GCS, Glasgow Coma Scale; NIHSS, National Institutes of Health Stroke Scale; ALT, alanine aminotransferase; AST, aspartate aminotransferase; eGFR, estimated glomerular filtration rate; FBG, fasting blood glucose*.

### Correlations Between ALP Levels and Clinical Outcomes

The incidences of the 30-day, 90-day, and 1-year poor functional outcomes were 61.4, 53.4, and 45.3%, respectively, among the highest ALP quartile. Compared with patients in the third quartile of ALP, the adjusted odds ratio of the highest quartile (>94.8 U/L) was 2.16 (1.32–3.55) for the 30-day poor functional outcome, 1.86 (1.12–3.10) for the 90-day poor functional outcome, and 2.26 (1.34–3.80) for 1-year poor functional outcome. However, a serum ALP in the lowest quartile ( ≤ 58.0 U/L) was not significantly correlated with 30-day, 90-day, and 1-year poor functional outcomes (Table 2). Subgroup analysis showed that age and sex had no interaction effect on the association between ALP levels and poor functional outcomes, although some ORs were significant in subgroups (Table 3).

**Table 2:** Crude and adjusted OR of ALP levels for 30-day, 90-day, and 1-year poor outcomes.

	**Q1 (≤58.0)**	**Q2 (58.0–75.0)**	**Q3 (75.0–94.8)**	**Q4 (>94.8)**
**30-day poor outcome**
Events, *n* (%)	138 (58.7)	129 (55.1)	120 (51.3)	145 (61.4)
Crude OR (95% CI)	1.35 (0.94–1.95)	1.17 (0.81–1.68)	1.00 (reference)	1.51 (1.05–2.18)
Adjusted^a^ OR (95% CI)	1.31 (0.80–2.15)	1.16 (0.71–1.89)	1.00 (reference)	2.16 (1.32–3.55)
**90-day poor outcome**
Events, *n* (%)	121 (51.5)	115 (49.2)	107 (45.7)	126 (53.4)
Crude OR (95% CI)	1.26 (0.88–1.81)	1.15 (0.80–1.65)	1.00 (reference)	1.36 (0.95–1.95)
Adjusted^a^ OR (95% CI)	1.15 (0.69–1.92)	1.14 (0.69–1.89)	1.00 (reference)	1.86 (1.12–3.10)
**1-year poor outcome**
Events, *n* (%)	110 (46.8)	102 (43.6)	84 (35.9)	107 (45.3)
Crude OR (95% CI)	1.57 (1.09–2.28)	1.38 (0.95–2.00)	1.00 (reference)	1.48 (1.02–2.15)
Adjusted^a^ OR (95% CI)	1.54 (0.92–2.59)	1.61 (0.96–2.70)	1.00 (reference)	2.26 (1.34–3.80)

*ALP, alkaline phosphatase; OR, odd ratios; CI, confidence interval.*

a *Adjusted for age, sex, alcohol, hypertension, diabetes mellitus, dyslipidemia, history of cerebral infarction, prior antiplatelet use, prior anticoagulant use, BMI, systolic blood pressure, diastolic blood pressure, GCS score, NIHSS score, location of hematoma, hematoma volume, ALT levels, AST levels, eGFR, fasting blood glucose, surgical treatment, and whether breaking into ventricle or subarachnoid*.

**Table 3:** Multivariate-adjusted OR and 95% CI for poor outcome according to quartiles of ALP levels, stratified by age and sex.

**Outcome**	**Subgroup**	**Q1 (≤58.0)**	**Q2 (58.0–75.0)**	**Q3 (75.0–94.8)**	**Q4 (>94.8)**	***p* for interaction**
30-day poor outcome	Age					0.46
	<70	1.41 (0.80–2.46)	1.31 (0.76–2.26)	1.00 (reference)	2.14 (1.24–3.69)	
	≥70	1.35 (0.38–4.81)	0.74 (0.22–2.51)	1.00 (reference)	4.20 (1.0–17.54)	
	Sex					0.23
	Male	1.29 (0.71–2.35)	0.99 (0.55–1.81)	1.00 (reference)	2.63 (1.43–4.82)	
	Female	1.59 (0.60–4.22)	1.70 (0.66–4.36)	1.00 (reference)	1.69 (0.65–4.36)	
90-day poor outcome	Age					0.71
	<70	1.10 (0.61–1.98)	1.22 (0.69–2.17)	1.00 (reference)	1.93 (1.10–3.40)	
	≥70	1.66 (0.44–6.18)	1.08 (0.30–3.94)	1.00 (reference)	2.77 (0.69–11.09)	
	Sex					0.57
	Male	1.11 (0.59–2.07)	1.13 (0.61–2.11)	1.00 (reference)	2.15 (1.15–4.00)	
	Female	1.55 (0.57–4.21)	1.34 (0.51–3.52)	1.00 (reference)	1.62 (0.61–4.25)	
1-year poor outcome	Age					0.57
	<70	1.46 (0.80–2.67)	1.37 (0.75–2.49)	1.00 (reference)	2.46 (1.37–4.41)	
	≥70	2.87 (0.75–10.96)	2.39 (0.64–8.92)	1.00 (reference)	1.60 (0.39–6.48)	
	Sex					0.08
	Male	1.26 (0.68–2.34)	1.36 (0.74–2.51)	1.00 (reference)	2.49 (1.34–4.60)	
	Female	2.61 (0.85–7.98)	2.37 (0.78–7.22)	1.00 (reference)	1.53 (0.49–4.83)	

*Adjusted for age, sex, alcohol, hypertension, diabetes mellitus, dyslipidemia, history of cerebral infarction, prior antiplatelet use, prior anticoagulant use, BMI, systolic blood pressure, diastolic blood pressure, GCS score, NIHSS score, location of hematoma, hematoma volume, ALT levels, AST levels, eGFR, fasting blood glucose, surgery, and whether breaking into ventricle or subarachnoid.*

*ALP, alkaline phosphatase; OR, odds ratio; CI, confidence interval*.

## Discussion

In this prospective cohort study of patients with ICH, higher ALP levels were correlated with increased risk of 30-day, 90-day, and 1-year poor functional outcomes, whereas no significant association was found between lower level of ALP and poor functional outcome. The association remained in the age and sex subgroups. Our findings suggest that the probability of high ALP concentration has an impact on the development and prognosis in patients with ICH.

Recently, the role of ALP has been highlighted in terms of its potential effects on various stroke outcomes. A large cohort study of patients with preserved kidney function suggested that higher serum ALP levels (>98 U/L) were connected with a 1.4-fold higher risk for 1-year all-cause mortality, stroke recurrence, composite endpoint, and poor functional outcome after stroke (12). Previous studies also suggested that a higher level of ALP was an independent prognostic factor for 3-month poor functional outcome and in-hospital mortality after acute cerebral infarction (17). Very limited research on the relationship between serum ALP and prognosis in patients with ICH has been carried out. A prospective study, including 221 ICH patients with a median follow-up period of 2.5 years, noted that a higher ALP (>97 U/L) was related to mortality rate after ICH (11). Findings from another prospective study in 639 patients indicated that elevated ALP (>96 U/L) was correlated with 30-day death and 90-day poor functional outcome after ICH (16). However, a prospective community-based study conducted in 10,754 participants with a median follow-up time of 16 years revealed that lower ALP levels were also related to increased risk of ischemic and hemorrhagic strokes (9). Different results in the literature may be explained by different study populations, sample size, and follow-up periods, and it is unclear whether both higher and lower ALP levels are correlated with poor prognosis after stroke onset. In our study, we found that higher ALP levels were associated with an increased risk of poor functional outcome in ICH patients, whereas no significant connection was observed between lower ALP levels and poor functional outcome.

Serum ALP levels might vary depending on sex (7). Shimizu et al. (9) noted that the associations of ALP levels and increased risk of stroke differed between males and females. The bone remodeling process, which could be regulated by ALP levels, increased in postmenopausal women due to estrogen deficiency (21, 22). Moreover, the study also suggested bone-type ALP expressed in vascular smooth muscle cells (10), so we assumed that the accelerated bone remodeling process might induce vascular dysfunction and further increase the risk of stroke. However, in our subgroup analysis, we did not discover the effects of ALP on poor outcome in ICH patients stratified by sex. This discrepancy might be due to the unbalanced proportion of females: 61.8% in the former study compared with 30.2% in our study. So further studies are needed to explore the sex difference in the relationships between ALP and ICH outcomes. In addition, the relative lower percentages of females in this cohort might partially reveal that women may be less likely to suffer from ICH in China, which was consistent with research findings conducted in Asian populations (23–25). However, studies from most of the Western countries have demonstrated that the incidence of ICH was comparable for males and females (26, 27). This phenomenon could be possibly explained by the higher prevalence of uncontrolled hypertension, smoking, and alcohol consumption observed in Asian men (3, 24, 26, 28, 29).

Several different mechanisms underlying the correlation of high serum ALP with poor functional outcome after ICH onset may be considered. First of all, ALP is often used as an early indicator of vascular calcification (15, 30, 31). Vascular calcification can further contribute to the process of atherosclerosis, which in turn results in vascular aging and increases the risk and extent of vessel rupture after stroke (7, 11, 16, 18). Moreover, studies indicated that vascular calcification especially occurring in intracranial internal carotid artery was an independent risk factor for hematoma enlargement (32) and closely related to deep cerebral microbleeds (33), which may result in poor outcomes after ICH. Secondly, serum ALP is identified as a surrogate marker of systematic inflammation (17, 34, 35). Higher levels of ALP may deteriorate the secondary cascade of injury associated with inflammation after ICH, which contribute to poor prognosis. Thirdly, ALP may be a reflection of malnutrition (12, 17). Increased ALP levels were associated with lower serum albumin levels and increased risk of infection-related mortality (11), contributing to adverse clinical outcomes in ICH patients. In addition, ALP may play an important role in principal functions of neural stem cells such as proliferation and differentiation (36–39). Neural progenitors have been found to express ALP and experiments *in vitro* have revealed that ALP knockdown reduces progenitor cell proliferation and differentiation (37, 38). Moreover, ALP has an impact on the process of axonal development (38, 40). Studies have shown that extracellular adenosine triphosphate (ATP) could repress axon growth and branching *via* the activation of P2X7 receptor (38, 40). ALP is able to hydrolyze the extracellular ATP and thus inhibit the activation of P2X7 receptor (38, 40). In this way, ALP could promote axonal growth. However, when ICH occurs, disruption of the neurons induced by hematomas and the damage of the blood–brain barrier (2) could further lead to the release of ALP expressed in neuronal membranes (41) into the plasma. Therefore, we speculate that higher serum ALP levels at the acute phase may reflect severe deficiency of ALP expressed in the brain and, thus, have an adverse effect on functions of neural stem cells and axonal development during the process of recovery after ICH, which leads to poor prognosis. The possible mechanisms underlying the relationship between lower ALP levels and poor ICH prognosis may be associated with impaired vascular homeostasis (9). The hematopoietic stem cells, which play a significant role in the maintenance of vasculature (42, 43), can be regulated by the activity of osteoblasts (44), and bone-type ALP expression, regulating the activity of osteoblasts, might further have an impact on the hematopoietic stem cells (9). Thus, lower ALP levels may be associated with impaired vascular homeostasis, which leads to unstable vasculature and a higher susceptibility to poor prognosis. Further analysis of the association between lower ALP levels and poor outcomes should be explored. These findings indicate that it is crucial to maintain optimal serum ALP levels for preventing poor prognosis after hemorrhagic stroke. Although in our study the increased risk did not reach statistical significance in the lowest quartile, we provide a new insight to better understand the association between serum ALP and poor functional outcomes in ICH patients.

There are some limitations in the present study. First, we recruited patients within 72 h after ICH onset and excluded more than 10% total number of patients lacking ALP levels and follow-up information. Given that deaths occurring within the first 48 h have been reported to be 11.3–27.5% (45–48) and patients with severe neurological deficits and larger hematoma may have not undergone the subsequent ALP testing and telephone follow-ups, our study exclude the most severe patients to some extent. Moreover, the majority of patients enrolled in our analysis were Han. Thus, the selection bias might exist and could have further limited the generalizability of our results to a population with more severe conditions and broader ethnic diversity. Secondly, around one-fifth of the patients in our research underwent surgery, which may have an impact on our analysis of the clinical outcomes. However, the Surgical Trial in Intracerebral Hemorrhage (STICH) trial and the Minimally Invasive Surgery Plus Alteplase in Intracerebral Hemorrhage Evacuation (MISTIE III) trials demonstrated that surgical treatment or minimally invasive surgery did not significantly improve the favorable functional outcome in patients with ICH (49–52). These findings need to be verified in further studies. Thirdly, the ALP isozymes were not tested in our study, so we could not evaluate which types of ALP were correlated with poor functional outcomes of ICH. Fourth, we only examined ALP levels at the acute period of ICH, but did not measure it before the onset of ICH or during hospitalization and the follow-up period. Thus, it is unknown whether the acute phase reaction accompanying ICH may influence ALP levels and whether changes of ALP levels may, in turn, have an impact on ICH outcomes. In addition, patients with elevated ALP levels had increased liver enzymes, indicating serum ALP levels could reflect hepatocellular injury (53, 54). Therefore, the presence of liver disease may influence our results. Although we excluded patients with liver failure and serum ALP levels still remained significantly associated with worse clinical outcomes after further adjustment for liver enzymes, limited information on systematic ultrasound examination for detecting subclinical liver disease could affect our results. Other potential factors such as dietary intake of vitamin D or related obstructive biliary diseases not collected in our study may have some residual confounding effect. Finally, a validation cohort might be needed to test and evaluate the efficiency of the ALP levels to predict the clinical outcomes in patients with ICH in our future analysis.

In conclusion, our results demonstrated that a high ALP level (>94.8 U/L) was independently associated with 30-day, 90-day, and 1-year poor functional outcomes in patients with ICH. Serum ALP might serve as a predictor for poor functional outcomes after ICH onset.

## Data Availability Statement

The raw data supporting the conclusions of this article will be made available by the authors, without undue reservation.

## Ethics Statement

The studies involving human participants were reviewed and approved by Institutional Review Board (IRB) of Beijing Tiantan Hospital. The patients/participants provided their written informed consent to participate in this study. Written informed consent was obtained from the individual(s) for the publication of any potentially identifiable images or data included in this article.

## Author Contributions

SL analyzed and interpreted the data and drafted the original manuscript. WW designed the research. QZ and YW analyzed and interpreted the data. AW conducted the statistical analyses. XZ designed the research and handled funding and supervision. All authors contributed to the article and approved the submitted version.

## Conflict of Interest

The authors declare that the research was conducted in the absence of any commercial or financial relationships that could be construed as a potential conflict of interest.

## Publisher's Note

All claims expressed in this article are solely those of the authors and do not necessarily represent those of their affiliated organizations, or those of the publisher, the editors and the reviewers. Any product that may be evaluated in this article, or claim that may be made by its manufacturer, is not guaranteed or endorsed by the publisher.

## Abbreviations

ICH: intracerebral hemorrhage
ALP: alkaline phosphatase
IRB: Institutional Review Board
BMI: body mass index
GCS: Glasgow Coma Scale
NIHSS: National Institutes of Health Stroke Scale
mRS: modified Rankin Scale score
ALT: alanine aminotransferase
AST: aspartate aminotransferase
FBG: fasting blood glucose
TC: total cholesterol
TG: triglyceride
LDL-C: low-density lipoprotein cholesterol
HDL-C: high-density lipoprotein cholesterol
eGFR: estimated glomerular filtration rate
SD: standard deviation
IQR: interquartile range
CI: confidence interval
OR: odds ratio.
