# Supplementary material for: Association Between Alkaline Phosphatase and Clinical Outcomes in Patients With Spontaneous Intracerebral Hemorrhage
Source: Front Neurol. 2021 Aug 30;12:677696. doi: 10.3389/fneur.2021.677696 (PMC8435581; doi:10.3389/fneur.2021.677696)
Supplement: Supplementary file 1 [file Table_1.DOCX]

Supplementary Table 1. Baseline characteristics and their univariate between included and excluded participants

|  | included  n=939 | excluded  n=1025 | P value |
| --- | --- | --- | --- |
| Male, n (%) | 655 (69.8) | 672 (65.6) | 0.05 |
| Age (years) | 58.7±13.2 | 55.1±15.1 | ＜0.0001 |
| Ethnic Han, n (%) | 905 (96.4) | 899 (87.7) | ＜0.0001 |
| Current smoking, n (%) | 307 (32.7) | 321 (31.3) | 0.51 |
| Alcohol, n (%) | 352 (37.5) | 364 (35.5) | 0.36 |
| Hypertension, n (%) | 896 (95.4) | 924 (90.2) | ＜0.0001 |
| Diabetes mellitus, n (%) | 323 (34.4) | 239 (23.3) | ＜0.0001 |
| Dyslipidemia, n (%) | 308 (32.8) | 218 (21.3) | ＜0.0001 |
| History of cerebral infarction, n (%) | 135 (14.4) | 132 (12.9) | 0.33 |
| Prior antiplatelet use, n (%) | 152 (16.2) | 125 (12.2) | 0.01 |
| Prior anticoagulant use, n (%) | 11 (1.2) | 10 (1.0) | 0.67 |
| BMI | 25.6±3.6 | 25.4±4.1 | 0.47 |
| SBP (mmHg) | 163.5 (149.0-185.0) | 165.0 (147.0-186.0) | 0.65 |
| DBP (mmHg) | 97.0 (84.0-109.0) | 95.0 (82.0-108.0) | 0.13 |
| GCS score | 14 (11-15) | 13 (6-15) | ＜0.0001 |
| NIHSS score | 10 (3-16) | 12 (4-25) | ＜0.0001 |
| Location of hematoma, n (%) |  |  | ＜0.0001 |
| lobar | 161 (17.2) | 140 (13.7) |  |
| deep | 585 (62.3) | 409 (39.9) |  |
| infratentorial | 93 (9.9) | 102 (10.0) |  |
| Hematoma volume (ml) | 14.6 (6.0-30.0) | 14.6 (8.0-40.6) | 0.0003 |
| Break into ventricle, n (%) | 311 (33.1) | 344 (33.6) | 0.84 |
| Break into subarachnoid, n (%) | 96 (10.2) | 168 (16.4) | ＜0.0001 |
| ALT (U/L) | 21.3 (14.0-31.0) | 26.1 (19.4-35.0) | ＜0.0001 |
| AST (U/L) | 21.0 (17.0-27.4) | 22.0 (18.0-28.9) | 0.0005 |
| eGFR (ml/min) | 54.4 (50.7-58.2) | 56.3 (52.1-60.4) | ＜0.0001 |
| FBG (mmol/l) | 5.9 (5.0-7.1) | 5.9 (5.9-5.9) | 0.97 |
| Surgery, n (%) | 200 (21.3) | 166 (16.2) | 0.0037 |

BMI: body mass index; SBP: systolic blood pressure; DBP: diastolic blood pressure; GCS: Glasgow Coma Scale; NIHSS: National Institutes of Health Stroke Scale; ALT: alanine aminotransferase; AST: aspartate aminotransferase; eGFR: estimated glomerular filtration rate; FBG: fasting blood glucose
